# Id3 expression identifies CD4^+^ memory Th1 cells

**DOI:** 10.1073/pnas.2204254119

**Published:** 2022-07-11

**Authors:** Laura A. Shaw, Tianda Z. Deng, Kyla D. Omilusik, Kennidy K. Takehara, Quynh P. Nguyen, Ananda W. Goldrath

**Affiliations:** ^a^Department of Biological Sciences, University of California, La Jolla, CA 92093

**Keywords:** CD4, Th1, memory, T cell

## Abstract

Memory CD4^+^ T cells play a pivotal role in mediating long-term protective immunity, positioning them as an important target in vaccine development. However, multiple functionally distinct helper CD4^+^ T-cell subsets can arise in response to a single invading pathogen, complicating the identification of rare populations of memory precursor cells during the effector phase of infection and memory CD4^+^ T cells following pathogen clearance and the contraction phase of infection. Furthermore, current literature remains unclear regarding whether a single CD4^+^ memory T-cell lineage gives rise to secondary CD4^+^ T helper subsets or if there are unique memory precursor cells within each helper lineage. A majority of T follicular helper (Tfh) cells, which have established memory potential, express Id3, an inhibitor of E protein transcription factors, following acute viral infection. We show that expression of Id3 definitively identified a subset of cells within both the CD4^+^ Tfh and T helper 1 (Th1) lineages at memory time points that exhibited memory potential, with the capacity for significant re-expansion in response to secondary infection. Notably, we demonstrate that a subset of Th1 cells that survive into the memory phase were marked by Id3 expression and possessed the potential for enhanced expansion and generation of both Th1 and Tfh secondary effector cell populations in a secondary response to pathogen. Additionally, these cells exhibited enrichment of key molecules associated with memory potential when compared with Id3^lo^ Th1 cells. Therefore, we propose that Id3 expression serves as an important marker to indicate multipotent potential in memory CD4^+^ T cells.

## Significance

Generation of a CD4^+^ T cell memory population is crucial for providing the rapid and more robust immune response following pathogen reexposure. Pinpointing memory CD4^+^ T cells among the multiple helper subsets generated in response to infection has been a challenge. However, given the role of memory T cells in protecting against reinfection, identifying this population is of significant interest. We found that the inhibitor of E protein transcription factors, Id3, was expressed by CD4^+^ memory T cells, allowing for the identification of memory CD4^+^ T cells with the ability to generate new T helper 1 (Th1) and T follicular helper (Tfh) subsets.

Generation of T-cell memory is crucial in conferring vaccine-induced immunity, particularly against pathogens where neutralizing antibodies alone are insufficient at providing long-term protection. Antigen-specific CD4^+^ T cells expand upon pathogen recognition and, depending on the infection milieu, differentiate into distinct effector cell types, including T helper 1 (Th1), Th2, Th17, T follicular helper (Tfh), and T regulatory (Treg) cells. Following the resolution of infection, a residual population of memory CD4^+^ T cells remains within the circulation or in tissues that persists long term and provides protection from reinfection (1). The memory CD4^+^ T-cell population within the circulation has conventionally been divided into two subsets: effector memory T (Tem) cells and central memory T (Tcm) cells (2, 3). Tem cells are defined by low expression of CD62L and CCR7, with access to nonlymphoid sites and the ability to produce effector cytokines within hours following T-cell receptor (TCR) stimulation. Tcm cells are characterized by high levels of CD62L and CCR7 and the ability to recirculate through lymph nodes, secrete interleukin-2 (IL-2) upon reactivation, and undergo significant proliferation to generate secondary effector CD4^+^ T cells (2).

Considerable efforts have been made to classify CD4^+^ T-cell memory precursor (MP) and memory T-cell populations based on expression of cell-surface receptors, transcription factors, and effector molecules such as cytokines. The fact that expression of many of these molecules occurs along a continuum rather than being polarized between subsets, compounded by the existence of lineage plasticity among the CD4^+^ T-cell subsets during primary and secondary responses, has added substantial complexity to this effort (4–8). Studies by several groups have attempted to relate unique phenotypic markers found on effector CD4^+^ T cells with their intrinsic potential to form long-lived memory cells (2, 9–11). Two prominent models have emerged: one positing that the MP and memory populations are heterogeneous, whereby each Th subset contains some cells that are long lived with expansion potential (11, 12), or, alternatively, one positing that the Tcm or Tfh subset serves as a unique source of memory CD4^+^ T cells, and a proportion of these cells are able to survive following the contraction phase to seed the memory T-cell compartment (9, 12).

The enriched multipotency of CXCR5^+^ Tfh memory cells (compared with CXCR5^−^) has been described following lymphocytic choriomeningitis virus (LCMV)–Armstrong (12–16). Using the Tem and Tcm paradigms for characterization, Pepper et al. found that CD4^+^ Tem (CXCR5^−^CCR7^−^) cells primarily gave rise to CXCR5^−^ (Th1) secondary effector cells, while Tcm (CXCR5^+^CCR7^+^) cells gave rise to both CXCR5^+^ (Tfh) and CXCR5^−^ (Th1) secondary effector cells in response to *Listeria monocytogenes* infection (2). Additional categorization into of memory CD4^+^ T cells into phenotypic subsets revealed a PSGL-1^hi^Ly6C^lo^ MP subset following acute LCMV infection that was shown to exhibit greater longevity and increased proliferation following antigen rechallenge compared with the PSGL-1^hi^Ly6C^hi^ subset (11). While the PSGL-1^hi^Ly6C^lo^ MP population was originally presumed to be primarily composed of Th1 cells, it was later suggested to also contain Tfh cells at a comparable frequency (9). Collectively, the evidence suggests that Th1 memory cells can persist and form secondary effector cells of only the Th1 lineage, while Tfh memory cells exhibit greater multipotency in the context of pathogen rechallenge. Additionally, formation of CD4^+^ Tcm phenotype cells was recently shown to require Thpok, which is also necessary for Tfh formation via suppression of Th1-associated transcription factors Blimp-1 and Runx3 (14). Therefore, it remains a question if the pluripotent memory CD4^+^ T-cell subset is necessarily contained within the Tfh CXCR5^+^CCR7^+^ population in all infection and inflammation contexts (17).

Despite clear differences between memory CD4^+^ and CD8^+^ T-cell populations (18), the model of CD8^+^ T-cell memory formation can serve as a valuable guiding framework for memory CD4^+^ T-cell investigations. Our laboratory and others have demonstrated the role of E and Id proteins in the differentiation of both short-lived effector and MP populations of CD8^+^ T cells (19–22). Notably, Id3 expression identified CD8^+^ T cells with memory potential at effector time points (19), which raises the possibility of an analogous role for Id3 in memory CD4^+^ T cells. E/Id proteins cooperate to regulate transcriptional programs necessary for Th-cell specification in naive, infection, and autoimmune settings (9, 16, 23–28); however, their role in differentiation and persistence of memory CD4^+^ T cells has not been studied as extensively. We found that a population of Id3^hi^ Th1 memory cells emerged following acute LCMV infection, which exhibited enhanced expansion potential and increased expression of memory-associated molecules such as CD127, Bcl2, and Tcf1 when compared with Id3^lo^ Th1 cells at memory time points. Relative to Id3^lo^ Th1 memory cells, we also found that Id3^hi^ Th1 cells exhibited a transcriptomic profile more akin to that of memory CD4^+^ T cells. Furthermore, while a majority of Th1 memory CD4^+^ T cells appeared limited in their ability to form both Th1 and Tfh secondary effectors, the Id3^hi^ Th1 memory CD4^+^ T cells presented as a small durable subset with enhanced multipotent recall potential. Therefore, we posit that Id3 expression serves as an important marker of multipotent memory CD4^+^ T cells.

## Results

### Helper CD4^+^ T Cells Share Transcriptomic Characteristics with Cytotoxic CD8^+^ T Cells.

To assess the possibility of common memory T-cell differentiation programs between CD4^+^ and CD8^+^ T cells, we compared global gene expression of effector and memory CD4^+^ SMARTA T cells (recognizing LCMV gp66-77 presented by major histocompatibility complex class II I-A^b^) with changes in gene expression in CD8^+^ T cells responding to LCMV-Armstrong infection. Strikingly, a majority of genes up-regulated by Th1 and Tfh subsets at days 7 and 41 following LCMV infection compared with naive SMARTA CD4^+^ T cells were those found within the effector or memory CD8^+^ T-cell gene signatures, respectively (*SI Appendix*, Fig. S1 *A* and *C*). Thus, despite biological differences among CD4^+^ and CD8^+^ T cells and Th1 versus Tfh populations, the two lineages shared unexpected similarities in gene expression at both effector and memory time points (*SI Appendix*, Fig. S1 *B* and *D*). Furthermore, gene set enrichment analysis (GSEA) indicated that the Tcm precursor (Tcmp) signature recently defined by Ciucci et al. (14) was enriched in both the Th1 and Tfh effector populations, suggesting that both of these lineages may harbor T-cell memory potential (*SI Appendix*, Fig. S1*E*).

### Id3-GFP–Expressing Memory CD4^+^ T Cells Expand and Give Rise to Th1 and Tfh Secondary Effector Cell Populations.

**Fig. 1.**
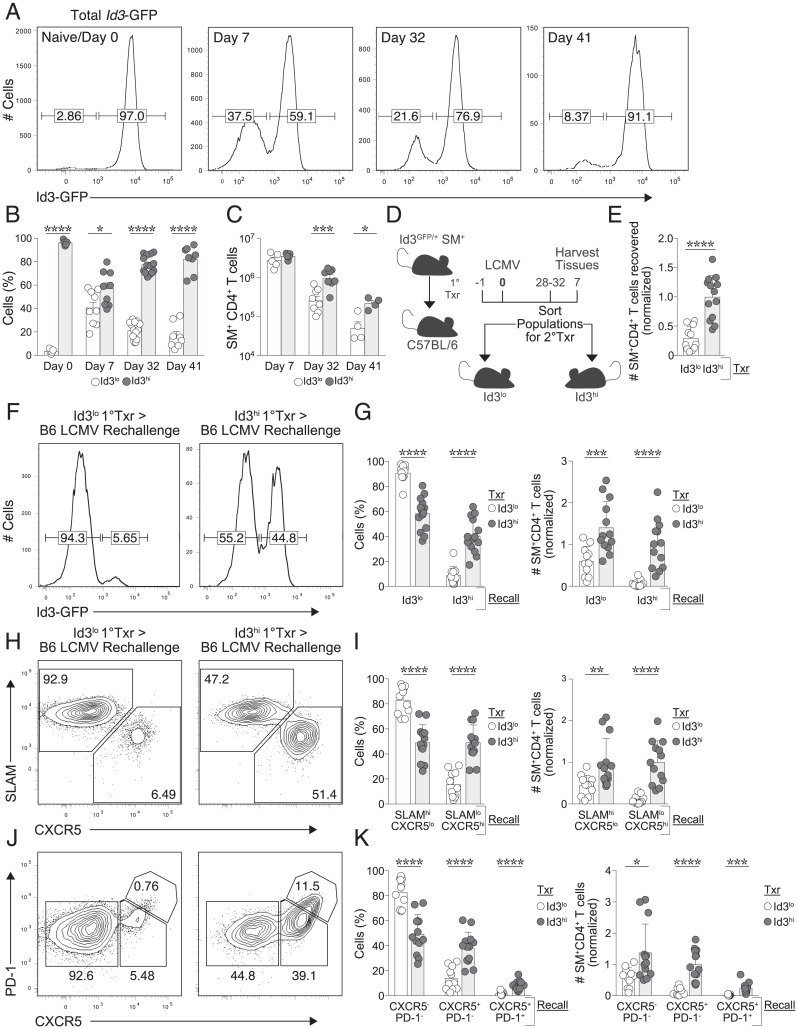
Id3 expression defines CD4^+^ T cells with increased memory potential. (*A*) Flow cytometric analysis of donor *Id3*^GFP/+^ SMARTA CD4^+^ T cells from C57BL/6 host mice over the course of an LCMV infection. (*B*, *C*) Frequency of Id3-expressing cells among SMARTA CD4^+^ T cells (*B*) or total SMARTA CD4^+^ T cells on indicated days of infection (*C*). (*D*–*K*) Id3-GFP^lo^ or Id3-GFP^hi^ memory SMARTA CD4^+^ T cells were sorted on days 28 to 32 (purity exceeded 99%) and transferred to naive C57BL/6 hosts that were then infected with LCMV to be analyzed 7/8 d later. (*D*) Experimental schematic for isolation of memory T cells based on expression of Id3. (*E*) Total SMARTA CD4^+^ T cells recovered from host mice at day 7/8 of secondary LCMV infection. (*F*) Analysis of Id3-GFP expression at day 7/8 of infection in donor cells from host mice that received transfers of either Id3-GFP^lo^ (left) or Id3-GFP^hi^ (right) memory SMARTA CD4^+^ T cells. Numbers on histogram peaks indicate percentage of cells within indicated gates. (*G*) Frequency among SMARTA CD4^+^ T cells (left) and total SMARTA CD4^+^ T cells (right) generated from indicated transferred populations in (*F*). (*H*) Analysis of the percentage of SLAM^hi^CXCR5^lo^ (Th1) cells or SLAM^lo^CXCR5^+^ (Tfh) cells generated from indicated memory populations following secondary infection. (*I*) Frequency among SMARTA CD4^+^ T cells (left) and total SMARTA CD4^+^ T cells (right) from indicated populations in (*H*) are shown. (*J*) Percentage of CXCR5^−^PD-1^−^ (Th1) cells, CXCR5^+^PD-1^−^ (Tfh) cells, or CXCR5^+^PD-1^+^ (GC Tfh) cells formed from indicated memory populations following secondary infection. (*K*) Frequency among SMARTA CD4^+^ T cells (left) and total SMARTA CD4^+^ T cells (right) from indicated populations in (*J*). *P < 0.05, **P < 0.01, ***P < 0.001, and ****P < 0.0001 (two-tailed unpaired Student’s *t* test). Data are representative of three experiments (*A*, *C*), each with n = 3 to 10 mice per group (mean ± SEM) or pooled from three (*B*, *E*–*K*) independent experiments with n = 3 to 10 mice per group (mean ± SEM).

Given the evident similarities in transcriptional signatures we observed between CD4^+^ and CD8^+^ T-cell populations, we hypothesized that, akin to CD8^+^ MP T cells, Id3 may serve as a marker of memory potential within effector CD4^+^ T-cell populations. We assessed kinetics of Id3 expression by adoptive transfer of CD4^+^ T cells using *Id3*^GFP/+^ reporter SMARTA TCR transgenic T cells (23). CD4^+^ T cells from these mice were transferred into congenically distinct hosts, which were infected 1 d later with LCMV. Consistent with our previous observations of high Id3 expression by naive CD8^+^ T cells (19), prior to infection more than 95% of CD4^+^ T cells expressed Id3-GFP (Fig. 1 *A* and *B*). Following infection, the proportion of effector CD4^+^ T cells with low Id3 expression significantly increased, but as the infection was cleared, upward of 90% of the remaining memory cells expressed Id3-GFP (Fig. 1 *A* and *B*), with a greater absolute number of Id3-expressing cells surviving the contraction phase and persisting to memory time points (Fig. 1*C*). Consistent with our previous studies, following infection, Id3-GFP^hi^ cells were almost exclusively Tfh (CXCR5^hi^SLAM^lo^), while a majority of Id3-GFP^lo^ cells displayed a Th1 phenotype (SLAM^hi^CXCR5^lo^) (*SI Appendix*, Fig. S2 *A* and *B*) (23). Since Id3 positive cells comprised a majority of the memory CD4^+^ T-cell population, we evaluated whether Id3-GFP^hi^ T cells had any advantages over Id3-GFP^lo^ memory cells in the context of reinfection. Id3-GFP^lo^ or Id3-GFP^hi^ SMARTA memory CD4^+^ T cells (28-32 d following primary infection) were transferred into a new cohort of B6 hosts, which were then infected 1 d later with LCMV (Fig. 1*D*). Following LCMV rechallenge, we found that both Id3-GFP^lo^ and Id3-GFP^hi^ SMARTA CD4^+^ T-cell populations were able to generate secondary effector Th cells. However, the phenotype and abundance of the expanded progeny were strikingly dissimilar (Fig. 1 *E*–*K*). We recovered 3.4-fold more secondary effector T cells derived from Id3-GFP^hi^ memory T cells than from Id3-GFP^lo^ memory cells (Fig. 1*E*), indicating that Id3-GFP^hi^ memory cells have significantly greater expansion potential. Secondary effector T cells generated from the transfer of Id3-GFP^lo^ memory cells also maintained low expression of Id3-GFP, whereas Id3-GFP^hi^ memory cells generated a mixed population of Id3-GFP^lo^ and Id3-GFP^hi^ secondary effector T cells (Fig. 1 *F* and *G*). A majority of secondary effector cells derived from Id3-GFP^lo^ memory cells were SLAM^hi^CXCR5^lo^ Th1 cells. Conversely, the Id3-GFP^hi^ memory T cells repopulated the CD4^+^ T-cell compartment with both Th1 (SLAM^hi^CXCR5^lo^) cells and Tfh (SLAM^lo^CXCR5^hi^) secondary effector T cells (Fig. 1 *H* and *I*). Furthermore, Id3-GFP^hi^ cells also generated a higher frequency of PD-1^+^CXCR5^+^ germinal center (GC) Tfh cells when compared with Id3-GFP^lo^ cells (Fig. 1 *J* and *K*). These data suggest that Id3-GFP–expressing memory T cells have enhanced expansion and multipotent recall potential and are capable of differentiating into both Th1 and Tfh cells upon rechallenge.

### Id3-GFP^hi^ Th1 Memory Cells Accumulate at Memory Time Points.

**Fig. 2.**
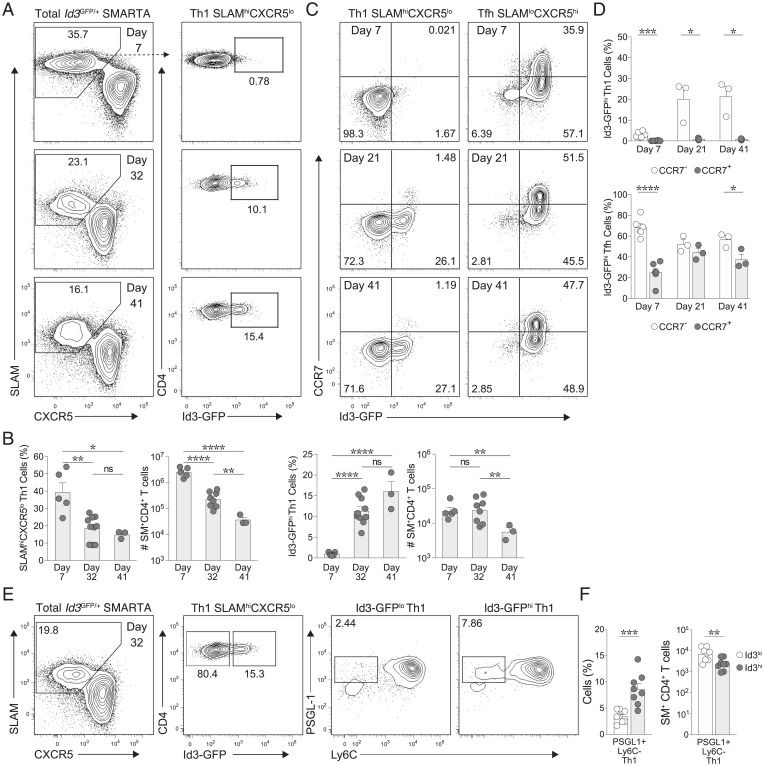
The Th1 population contains Id3-expressing cells. (*A*) Flow cytometric analysis of donor *Id3*^GFP/+^ SMARTA CD4^+^ T cells from C57BL/6 host mice at indicated day of LCMV infection (7, 32, 41). Numbers in outlined area indicate percentage of SLAM^hi^CXCR5^lo^ (Th1) cells (left) and expression of Id3-GFP within the Th1 compartment (right). (*B*) Frequency of SLAM^hi^CXCR5^lo^ (Th1) cells (far left) and total SMARTA CD4^+^ T cells (second from left); frequency of Id3-GFP-expressing cells among SLAM^hi^CXCR5^lo^ (Th1) cells (second from right) and total SMARTA CD4^+^ T cells (far right). (*C*) Analysis of CCR7 expression on *Id3*^GFP/+^ SMARTA CD4^+^ Th1 and Tfh cells at days 7, 21, and 41 of LCMV infection. (*D*) Frequency of Id3^+^CCR7^−^ and Id3^+^CCR7^+^ cells among Th1 (top) and Tfh (bottom) cells. (*E*) Flow cytometric analysis of donor *Id3*^GFP/+^ SMARTA CD4^+^ T cells from C57BL/6 host mice at day 32 of LCMV infection. Percentage of Id3-GFP expression on SLAM^hi^CXCR5^lo^ (Th1) and SLAM^lo^CXCR5^hi^ (Tfh) cells, with subsequent PSGL-1 and Ly6C analysis from Id3-GFP^hi^ or Id3-GFP^lo^ Th1 and Id3-GFP^hi^ Th1. (*F*) Frequency among SMARTA CD4^+^ T cells (left) and total SMARTA CD4^+^ T cells (right) from indicated populations in (*E*). *P < 0.05, **P < 0.01, ***P < 0.001, and ****P < 0.0001 (two-tailed unpaired Student’s *t* test). Data are representative of two experiments each with n = 3 to 8 mice per group (mean ± SEM). ns, not significant.

Both Th1 and Tfh memory T cells persist following LCMV infection (Fig. 2*A*), but we observed a decrease in the frequency of Th1 (CXCR5^−^) cells over time. Within this waning population, however, we observed the emergence of an Id3-GFP–expressing memory Th1 population, where ∼15% of Th1 memory cells expressed Id3-GFP by day 41 following infection (Fig. 2 *A* and *B*). While the frequency of Id3-GFP–expressing Th1 cells increased as the total Th1 population contracted, the absolute number of Id3-GFP–expressing Th1 cells was evident as early as day 7 of infection and was mainted into the memory timepoint (Fig. 2 *A* and *B*). Tcm CD4^+^ T cells possess enhanced differentiation potential and are traditionally marked by CCR7 expression. To assess how this small population of Id3-GFP^hi^ Th1 memory cells might factor into the broader paradigm of Tcm and Tem CD4^+^ populations, we analyzed the expression of CCR7 by this Id3-GFP–expressing Th1 memory population (2, 3, 11). While a significant portion of Id3-GFP^hi^ Tfh memory cells exhibited CCR7 expression (Fig. 2 *C* and *D*), Id3-GFP^hi^ Th1 memory cells did not gain expression of CCR7 (Fig. 2 *C* and *D*), suggesting that they do not fit the canonical Tcm-cell criteria.

We further interrogated characteristics of these cells through expression of PSGL-1 and Ly6C. Historically, these surface markers, in combination with T-bet, have been used to identify unique subsets of antigen-specific CD4^+^ T cells: PSGL-1^hi^Ly6C^hi^T-bet^hi^ Th1 (terminally differentiated), PSGL-1^hi^Ly6C^lo^-T-bet^int^ Th1 (higher recall capacity and able to convert to T-bet^hi^), and PSGL-1^lo^Ly6C^lo^T-bet^lo^ Tfh cells (11). We observed that the frequency of PSGL-1^hi^Ly6C^lo^-bet^int^ Id3-GFP^hi^ Th1 cells was increased 2.5-fold compared with the Id3-GFP^lo^ Th1 compartment (Fig. 2 *E* and *F*). Thus, by multiple parameters, Id3-GFP^hi^ Th1 cells displayed characteristics of Th1 memory cells.

### Id3-GFP^hi^ Th1 Memory Cells Give Rise to Th1 and Tfh Cells in a Secondary Response.

**Fig. 3.**
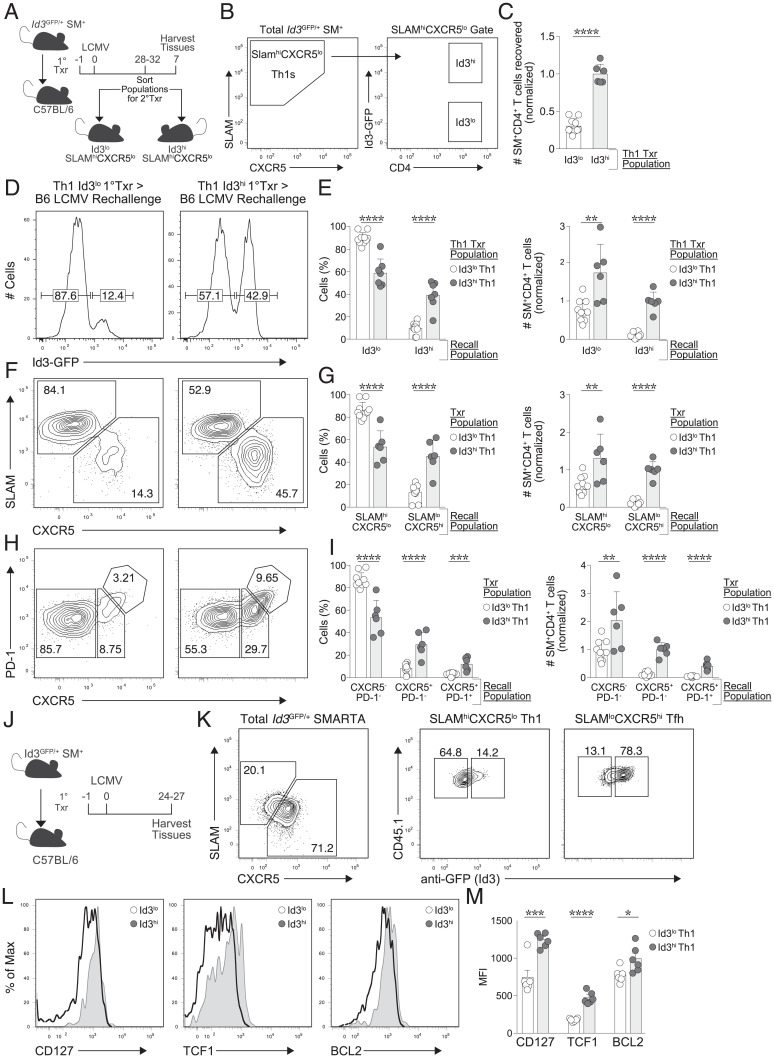
Id3-GFP^hi^ Th1 memory cells exhibit increased accumulation and multipotency upon rechallenge. (*A*–*I*) C57BL/6 host mice received a transfer of either Id3-GFP^lo^ or Id3-GFP^hi^ SLAM^hi^CXCR5^lo^ Th1 memory SMARTA CD4^+^ T cells and were infected with LCMV for 7/8 d before analysis. (*A*, *B*) Experimental schematic for sorting SLAM^hi^CXCR5^lo^ Th1 cells based on expression of Id3 (purity exceeded 99%). (*C*) Total SMARTA CD4^+^ T cells recovered from Id3^lo^ Th1 or Id3^hi^ Th1 secondary transfer on day 7/8 of reinfection. (*D*) Expression of Id3 in indicated memory populations on day 7/8 of reinfection. (*E*) Frequency among SMARTA CD4^+^ T cells (left) and total SMARTA CD4^+^ T cells (right) generated from indicated transferred populations in (*D*). (*F*) Percentage of SLAM^hi^CXCR5^lo^ (Th1) cells or SLAM^lo^CXCR5^+^ (Tfh) cells generated from indicated memory populations on day 7/8 of reinfection. (*G*) Frequency among SMARTA CD4^+^ T cells (left) and total SMARTA CD4^+^ T cells (right) from indicated populations in (*F*). (*H*) Percentage of CXCR5^−^PD-1^−^ (Th1) cells, CXCR5^+^PD-1^−^ (Tfh) cells, or CXCR5^+^PD-1^+^ (GC Tfh) cells formed from indicated memory populations at day 7/8 of reinifection. (*I*) Frequency among SMARTA CD4^+^ T cells (left) and total SMARTA CD4^+^ T cells (right) from indicated populations in (*H*). (*J*–*M*) Analysis of memory Id3^GFP/+^ SMARTA CD4^+^ T cells at day 30 of LCMV infection. (*J*) Schematic of experimental timeline. (*K*) Frequency of SLAM^hi^CXCR5^lo^ (Th1) cells or SLAM^lo^CXCR5^hi^ (Tfh) cells among total donor cells (right) and Id3 expression in indicated donor subset (right). (*L*) Histograms show expression of indicated protein on Id3-GFP^lo^ (black) or Id3-GFP^hi^ (gray shaded) Th1 memory populations. (*M*) Quantification of median fluorescence intensity for indicated protein. *P < 0.05, **P < 0.01, ***P < 0.001, and ****P < 0.0001 (two-tailed unpaired Student’s *t* test). Data are normalized and pooled from two independent experiments with n = 3 to 10 mice per group (mean ± SEM). MFI, mean fluorescence intensity.

As we previously found that the Id3-GFP^hi^ memory T-cell population exhibited greater multipotent potential during secondary challenge, we next evaluated whether this was the case specifically within the Th1 memory lineage. Id3-GFP^hi^ and Id3-GFP^lo^ Th1 primary memory cells were sorted and transferred into a new cohort of B6 hosts, which were infected 1 d later with LCMV (Fig. 3 *A* and *B*). Sort purity for each parameter exceeded 99% (*SI Appendix*, Fig. S2*C*). Following reinfection, we found that Id3-GFP^lo^ Th1 donors primarily generated secondary effector cells with low expression of Id3-GFP, whereas Id3-GFP^hi^ Th1 donors were able to generate 3.3-fold more secondary effector cells with a mixed population of both Id3-GFP^lo^ and Id3-GFP^hi^ cells (Fig. 3 *D*–*I*). Secondary effector T cells from Id3-GFP^lo^ Th1 donors were predominantly Th1 cells, while secondary effector T cells from Id3-GFP^hi^ Th1 donors were composed of both Th1 (SLAM^hi^CXCR5^lo^) cells and Tfh (SLAM^lo^CXCR5^hi^) cells (Fig. 3 *F* and *G*). Furthermore, Id3-GFP^hi^ Th1 cells also generated a higher frequency of PD-1^+^CXCR5^+^ GC Tfh cells when compared with Id3-GFP^lo^ Th1 cells (Fig. 3 *H* and *I*). Collectively, the data suggest that Id3-GFP^hi^ Th1 memory cells have greater multipotent recall potential compared with Id3-GFP^lo^ Th1 memory cells, despite the absence of CCR7 expression and canonical Tcm phenotype (3).

We further characterized the Id3-GFP^hi^ and Id3-GFP^lo^ Th1 memory populations for expression of key molecules associated with long-lived memory T cells. Notably, Id3-GFP^hi^ Th1 memory cells expressed significantly more IL-7 receptor (CD127), suggesting a greater responsiveness to IL-7 that would promote memory T-cell survival and homeostasis (29–31). Correspondingly, Id3-GFP^hi^ Th1 cells expressed increased levels of the antiapoptotic molecule BCL2, further supporting the notion that Id3-GFP^hi^ Th1 cells have an increased capacity for survival compared with their Id3-GFP^lo^ counterparts. Finally, TCF1, a transcription factor important for memory CD8^+^ T-cell formation and function (32, 33) and Tfh development (34–36), was expressed at higher levels in Id3-GFP^hi^ Th1 memory cells compared with the Id3-GFP^lo^ Th1 memory population (Fig. 3 *J*–*M*). These data suggest that expression of Id3-GFP imbues a population of Th1 memory cells with enhanced memory T-cell characteristics, including greater survival, expansion, and multipotent differentiation potential.

### Id3 Expression Defines a Transcriptionally Distinct Th1 Memory Population.

**Fig. 4.**
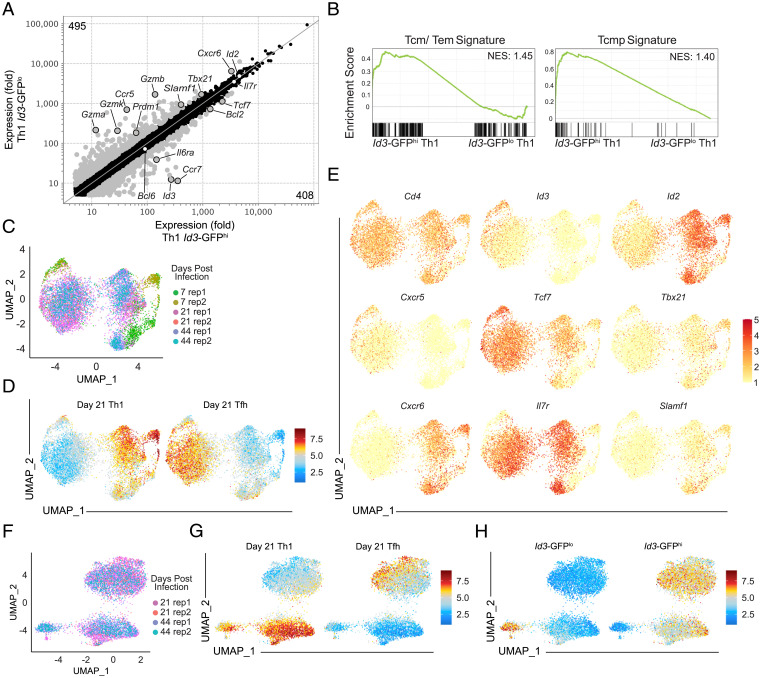
Id3-GFP^hi^ cells are a transcriptionally distinct Th1 memory cell subset. (*A*) Averaged (three independent replicates) messenger RNA expression by expression plot of Th1 Id3-GFP^hi^ versus Id3-GFP^lo^ cells from bulk RNA-seq. Highlighted genes (gray) indicate fold change ≥1.75. (*B*) GSEA of Tcm (41) and Tcmp (14) signatures in memory CD4^+^ Id3-GFP^hi^ Th1 versus Id3-GFP^lo^ Th1 cells. (*C*–*G*) SMARTA CD4^+^ T cells were adoptively transferred into congenically distinct hosts 1 d before infection with LCMV. Splenocytes were harvested and SMARTA CD4^+^ T cells were sorted at 7, 21, and 41 d of infection and subsequently processed for scRNA-seq with the 10× Genomics platform. (*C*) UMAP plot of samples colored by sample ID. (*D*) UMAP plot of relative enrichment of memory Th1 (left) and Tfh (right) gene signatures generated from bulk RNA-seq of sorted Tfh and Th1 memory cells. (*E*) Relative expression of indicated genes, including known Th1- and Tfh-associated genes. (*F*) UMAP plot of samples colored by sample ID. (*G*) Relative enrichment of memory Th1 (left) and Tfh (right) gene expression signatures generated from bulk RNA-seq of sorted Tfh and Th1 memory cells. (*H*) Relative enrichment of Th1 Id3-GFP^lo^ (left) and Id3-GFP^hi^ (right) memory gene expression signatures generated from bulk RNA-seq. NES, normalized enrichment score.

To examine the transcriptional differences between Id3-GFP^lo^ and Id3-GFP^hi^ Th1 memory cells, we performed RNA sequencing (RNA-seq) on these sorted populations (day >30 of LCMV infection). Id3-GFP^hi^ Th1 memory cells were enriched for transcripts encoding key memory genes, including *Bcl2* and *Tcf1*, compared with Id3-GFP^lo^ Th1 memory cells (Fig. 4*A*). Relative to their Id3-GFP^hi^ counterparts, Id3-GFP^lo^ Th1 memory cells were enriched for effector molecule transcripts (*Prdm1*, *Gzma*, *Gzmb*, and *Gzmk*), suggesting a more effector-like transcriptional profile (Fig. 4*A*), whereas expression of *Id2* and *Bcl6* were equivalent, confirming their Th1 identity. When directly comparing Id3-GFP^hi^ Th1 memory cells with Id3-GFP^hi^ Tfh memory cells, Id3-GFP^hi^ Th1 memory cells expressed Th1-associated transcripts compared with Id3-GFP^hi^ Tfh memory cells, emphasizing that these were distinct memory populations (*SI Appendix*, Fig. S3*A*). Notably, the Tfh memory population was also enriched for memory-associated genes (*SI Appendix*, Fig. S3*A*), suggesting that CD4^+^ T-cell memory subsets may persist along a cell-state continuum as previously described (8). Using GSEA, we found that when compared with Id3-GFP^lo^ Th1 cells, Id3-GFP^hi^ memory cells were significantly enriched for the Tcm memory signature (defined by up-regulated transcripts compared with Tem cells) as well as the Tcmp signature (14) (Fig. 4*B*).

As we observed marked phenotypic and functional differences between Id3-GFP^hi^ and Id3-GFP^lo^ Th1 memory cells, we investigated the heterogeneity within the CD4^+^ effector and memory T-cell populations by single-cell RNA-seq (scRNA-seq) of SMARTA CD4^+^ T cells at days 7, 21, and 41 of LCMV infection (Fig. 4). Unsupervised clustering with visualization via UMAP (uniform manifold approximation and projection) revealed distinct clusters of cells that correlated with Tfh and Th1 subsets based on expression of canonical lineage markers at each time point (Fig. 4 *C* and *E*). Memory T cell–associated genes (including *Tcf7* and *Il7r*) were enriched in the day-21 and day-41 samples, while key markers of the Tfh (*Cxcr5*) and Th1 (*Tbx21*, *Cxcr6*, *Slamf1*) lineages exhibited mutually exclusive enrichment in the clusters (Fig. 4*E*). To expand this analysis, we next defined the gene expression signature of Th1 and Tfh memory cells by performing bulk RNA-seq on sorted Th1 and Tfh memory SMARTA populations on day 21 of LCMV infection. We found that 2,325 (1,049 Tfh plus 1,276 Th1) genes were differentially expressed, with a fold change of ≥2 between these two populations (*SI Appendix*, Fig. S3*B*). We overlayed the Tfh and Th1 memory signatures onto the single-cell projections to identify individual cells enriched for expression of the Th1 or Tfh memory cell transcriptome (Fig. 4*D*). The Th1 signature–enriched cells showed expression of Id3 corresponding to the small population of Id3-GFP^hi^ Th1 memory cells identified in vivo that also expressed memory-associated genes, including *Tcf7*, *Bcl2*, and *Il7r* (*SI Appendix*, Fig. S3*E*). Notably, cells on day 7 of the response also showed moderate enrichment for our Th1 and Tfh signatures, consistent with the presence of an Id3^hi^ CD4^+^ memory precursor population as previously described in endogenous CD4^+^ T cells (14).

To understand how the Tcm versus Tem dichotomy broadly applies to memory CD4^+^ T cells, we overlayed Tcm and Tem signatures from memory CD8^+^ T-cell subsets onto the memory CD4^+^ T-cell scRNA-seq data (*SI Appendix*, Fig. S3*F*). Enrichment of the Tem signature correlated with day-7 effector cell samples as well as Th1 signature–enriched clusters, while the Tcm signature exhibited greater overlap with memory time point cells (days 21 and 44) as well as Tfh signature–enriched clusters. This analysis suggests that without prior division into subsets, Tfh memory cells overall exhibit a more Tcm-like phenotype than Th1 memory cells. The Tcmp signature (14) also showed enrichment in memory Th1 cells (cluster 2), supporting our observation that these cells are bona fide Th1 memory cells (*SI Appendix*, Fig. S3*G*).

To focus on the heterogeneity of memory CD4^+^ T cells specifically, we performed unsupervised clustering of memory time point cells (days 21 and 44), which revealed three major clusters (Fig. 4*F*). When we overlayed Th1 and Tfh memory signatures onto these data, we found the Tfh signature was enriched in one distinct cluster, while the Th1 signature spanned the remaining two clusters (Fig. 4*G*). To test whether the two Th1-enriched clusters represented Id3-GFP^hi^ and Id3-GFP^lo^ Th1 memory cells, we overlayed Th1 Id3-GFP^hi^ and Id3-GFP^lo^ memory signatures (defined by fold change >1.75) generated from bulk RNA-seq (Fig. 4*A*) onto the single-cell analyses. Indeed, the Th1 Id3-GFP^hi^ and Th1 Id3-GFP^lo^ memory signatures exhibited mutually exclusive polarization within Th1-enriched clusters, with the Th1 Id3-GFP^hi^ signature highlighting both Th1 and Tfh memory clusters (Fig. 4*H*). Taken together, the scRNA-seq data definitively showed that the Id3-GFP^hi^ Th1 population as a subset of long-lived memory CD4^+^ T cells was transcriptionally distinct from Tfh memory cells and Id3-GFP^lo^ Th1 cells.

## Discussion

The specific identity of the memory CD4^+^ T-cell population has historically been somewhat elusive. Contrary to their CD8^+^ T-cell counterparts, memory CD4^+^ T cells can be found at relatively low frequencies and decay following the contraction phase. However, given the role of CD4^+^ memory T cells in protecting against reinfection and expanded interest in the design of vaccines that elicit long-lived CD4^+^ Th-cell populations to support B-cell memory and host protection, efforts have increased toward defining this population. We investigated a previously unexplored role for Id3 in memory CD4^+^ T-cell potential.

We found that expression of the transcriptional regulator Id3 defined a transcriptionally distinct population of CD4^+^ T cells with enhanced memory potential. Importantly, within the Th1 compartment, we identified a subset of Id3-GFP^hi^ cells that appeared as early as day 7 of acute viral infection and accumulated in frequency as the Th1 compartment contracted but were consistent in numerical quantity over time. Compared with Id3-GFP^lo^ Th1 memory cells, Id3-GFP^hi^ Th1 memory cells exhibited greater multipotency and proliferative potential upon secondary challenge. Furthermore, Id3-GFP^hi^ Th1 memory cells showed increased expression of molecules critical for T-cell memory formation and survival when compared with Id3-GFP^lo^ memory Th1 cells. scRNA-seq revealed that Id3-GFP^hi^ Th1 memory cells formed a distinct population that retained genes associated with Th1 polarization while also up-regulating memory-associated molecules enriched in the Tcm and Tfh memory compartments.

Our data defined Id3^hi^SLAM^hi^CXCR5^lo^ as the subset of Th1 memory cells with stem-like properties, a characteristic previously associated primarily with CXCR5^hi^ Tfh memory CD4^+^ T cells. Considering these data in combination with our previous studies showing Id3 expression as a defining characteristic of SLAM^lo^CXCR5^hi^ memory CD4^+^ T cells (23), we have now established Id3 as a reliable marker for cells in both the Th1 and Tfh compartments, with the ability to demonstrate multipotent recall upon secondary challenge. The emergence of this Th1 population in frequency, along with the maintenance in cell number, is consistent with these memory cells originating from memory precursor cells in the Th1 population. However, it is also possible that a proportion of Tfh or uncommitted cells could give rise to these Th1 memory cells. Identifying universal markers of memory such as Id3 across T-cell subsets may serve to deconvolute the complexity associated with defining CD4^+^ T-cell memory and provide an opportunity for therapeutic potential in the future.

## Materials and Methods

### Mice.

All mice were housed under specific pathogen-free conditions in an American Association of Laboratory Animal Care–approved facility at the University of California, San Diego, CA (UCSD), and all procedures were approved by the UCSD Institutional Animal Care and Use Committee. Id3-GFP mice (37), SMARTA mice (38) (with transgenic expression of an I-A^b^–restricted TCR specific for LCMV glycoprotein amino acids 66 to 77), and recipient C57BL/6J mice were either bred at UCSD or received from The Jackson Laboratory.

### T-Cell Transfer and Infection.

Naive CD45.1^+^ or CD45.1.2^+^ SMARTA CD4^+^ T cells (25,000 cells per mouse) were adoptively transferred into congenically distinct wild-type C57BL/6J recipients 1 d before infection with 2 × 10^5^ plaque-forming units of LCMV-Armstrong, injected intraperitoneally.

### Cell Preparation and Flow Cytometry.

Single-cell suspensions of spleen were prepared by standard mechanical disruption. Surface staining for flow cytometry was performed with monoclonal antibodies against CD4 (RM4-5, 1:400), CD45.1 (A20, 1:400), CD45.2 (104, 1:400), B220 (RA3-6B2, 1:400), PD-1 (J43, 1:400), SLAM (TC15-12F12.2, 1:400), CD4 (GK1.5, 1:400), CD127 (A7R34, 1:400), and CCR7 (4B12, 1:200). Staining was done for 30 min at 4 °C in phosphate-buffered saline (PBS) supplemented with 0.5% bovine serum albumin and 0.1% sodium azide, unless specified otherwise. CCR7 staining was completed prior to other surface markers at 37 °C for 45 min. CXCR5 staining was performed using purified anti-CXCR5 (SPRCL5, 1:50; Invitrogen) for 30 min, followed by PE-Cy7– or BV510-labeled streptavidin (1:1,000; eBioscience) at 4 °C. Intracellular staining was performed with monoclonal antibodies to Bcl2 (clone 3F11, 1:20; BD Pharmingen), TCF1 (clone C63D9, 1:200; Cell Signaling), and polyclonal antibodies against GFP (cat. A21331; Invitrogen) using the Foxp3 ICS kit according to manufacturer’s instructions (eBioscience). Stained cells were analyzed using LSRII, LSRFortessa, or LSRFortessa X-20 (BD Biosciences) and FlowJo software (TreeStar). All sorting was completed on a FACSAria (BD Biosciences).

### Bulk RNA-Seq Library Construction and Sequencing.

Sorted cell lysates (5 μL) were used for Smart-seq2 library construction, prepared as previously described (39, 40) with slight modifications. Briefly, total RNA was captured and purified on RNAClean XP beads (Beckman Coulter). Polyadenylated messenger RNA was then selected using an anchored oligo(dT) primer (5′-AAGCAGTGGTATCAACGCAGAGTACT30VN-3′) and converted to complementary DNA (cDNA) via reverse transcription. First-strand cDNA was subjected to limited PCR amplification followed by Tn5 transposon–based fragmentation using the Nextera XT DNA Library Preparation Kit (Illumina). Samples were then PCR amplified for 12 cycles using barcoded primers such that each sample carried a specific combination of eight base Illumina P5 and P7 barcodes and pooled together prior to sequencing. Smart-seq paired-end sequencing was performed on an Illumina NextSeq500 (two full NextSeq runs per batch of 96 samples for 10 million raw reads per sample on average) using 2 × 38 bp reads with no further trimming.

### 10× Genomics Library Preparation and Sequencing.

Sorted cells were washed and resuspended in PBS and 0.04% (wt/vol) bovine serum albumin per the manufacturer’s guidelines. Single-cell libraries were prepared according to the protocol for 10× Genomics for single-cell V(D)J and 5′ gene expression. Approximately 10,000 sorted SMARTA cells were loaded and partitioned into Gel Bead In-Emulsions. scRNA libraries were sequenced on a HiSeq4000 (Illumina).

### scRNA-Seq Analysis.

scRNA-seq analysis was performed using Cell Ranger software and Seurat version 3.5.1 in R Studio. Cell Ranger was used with default parameters. Seurat analysis of 10× counter matrices was done by following these steps: low-quality cells, identified by percentage of mitochondria <10 and nFeatures_RNA <200 or >3,000, were removed; counts were normalized with FastMNN; and dimensionality reduction and cluster identification were done with UMAP (dims, 1:30), FindNeighbors (dims, 1:30), FindClusters (resolution, 0.6), and FindAllMarkers function with default parameters and min.pct of 0.25 and logfc.threshold of 0.25. Overlay of gene signatures onto single-cell data was done with AddModuleScore.

### Statistical Methods.

Statistical tests were performed using Prism version 7.0/9.0 (GraphPad). Significance was determined by unpaired Student’s *t* test with 95% confidence interval.

### Study Approval.

All animal studies were approved by the UCSD Institutional Animal Care and Use Committees and performed in accordance with UC guidelines.

## Supplementary Material

Supplementary File

## Data Availability

Sequencing data have been deposited in National Center for Biotechnology Information Gene Expression Omnibus repository under accession no. GSE175743 and can be accessed at https://www.ncbi.nlm.nih.gov/projects/geo/query/acc.cgi?acc=GSE175743.
