# Supplementary material for: Id3 expression identifies CD4+ memory Th1 cells
Source: Proc Natl Acad Sci U S A. 2022 Jul 11;119(29):e2204254119. doi: 10.1073/pnas.2204254119 (PMC9303986; doi:10.1073/pnas.2204254119)
Supplement: Supplementary File [file pnas.2204254119.sapp.pdf]

Supplemental Figure Legends

Figure S1. Shared transcriptional features between CD4$^+$ and CD8$^+$ memory T cells

(a) Volcano plots showing averaged mRNA expression (3-4 independent replicates) of Th1 (left) or Tfh (right) effector SMARTA CD4$^+$ T cells vs averaged mRNA expression (2 independent replicates) of naive SMARTA CD4$^+$ T cells. The effector CD8$^+$ T cell gene-expression signature defined by transcripts enriched in both terminal effector (TE) and memory precursor (MP) CD8$^+$ T cells over naive CD8$^+$ T cells (FC ≥ 2) from previous published data (Milnet et al., PNAS, 2020) (41) is overlaid (highlighted in orange). **(b)** GSEA of effector CD8$^+$ T cell gene-signature in effector vs naive CD4$^+$ T cells. **(c)** Volcano plots showing averaged mRNA expression (2 independent replicates) of Th1 (left) or Tfh (right) memory SMARTA CD4$^+$ T cells vs averaged mRNA expression (2 independent replicates) of naive SMARTA CD4$^+$ T cells. The memory CD8$^+$ T cell gene-expression signature defined by transcripts enrichment in both Tem and Tcm populations over naive CD8$^+$ T cells (FC ≥ 2) from previous published data (Milnet et al., PNAS, 2020) (41) is overlaid (highlighted in blue). **(d)** GSEA of memory CD8$^+$ T cell gene-signature in memory vs naive CD4$^+$ T cells. **(e)** GSEA of Tcmp signature in memory vs naive CD4$^+$ T cells.

Figure S2. Id3 expression defines CD4$^+$ T cell subsets

(a) Flow cytometric analysis of donor *Id3*$^{GFP/+}$ SMARTA CD4$^+$ T cells from C57BL/6 host mice on days 7 and 41 of an LCMV infection. Numbers in outlined area indicate percent SLAM^{hi}CXCR5^{lo} (Th1) and SLAM^{lo}CXCR5^{hi} (Tfh) within the Id3-GFP^{lo} (top) or Id3-GFP^{hi} (bottom) population. **(b)** Frequency among SMARTA CD4$^+$ T cells (top) and total SMARTA CD4$^+$ T cells (bottom) from indicated populations in (**a**) are shown. **(c)** Memory timepoint pre- and post-sort purity from hosts that received total *Id3*$^{GFP/+}$ referred to in Fig 3a,b, followed by LCMV infection. Post-sort Id3-GFP^{hi} and Id3-GFP^{lo} Th1 cell purity (top), and frequency of expression on the same cells. *$P < 0.05$, **$P < 0.01$, ***P<0.001 and ****$P < 0.0001$ (two-tailed unpaired Student′ s t test). Data are representative of 3 experiments, each with n= 3-10 mice per group (mean ± s.e.m.).

Figure S3. Single-cell RNA-seq and bulk-RNA sequencing of memory SMARTA CD4$^+$ T cells

(a) Averaged (3 independent replicates) mRNA expression by expression plot of > day 30 memory Tfh Id3-GFP^{hi} vs Th1 Id3-GFP^{hi}. Highlighted genes (grey) indicate fold change ≥1.75. **(b)** Averaged (4 independent replicates) mRNA expression by expression plot of day 21 Tfh vs Th1 memory cells. Highlighted genes (grey) indicate those of the Th1 (left) or Tfh (right) memory gene-signatures defined by FC ≥ 2. **(c)** uMAP plot of samples colored by cluster. **(d)** Violin plots of relative enrichment of memory Th1 (left) and Tfh (right) gene-signatures generated from bulk-

RNA sequencing of sorted Tfh and Th1 memory cells. **(e)** Relative expression of Id3 and indicated memory-associated genes on uMAP plots containing cells enriched for the Th1 memory gene-signature. For filtering, violin plots were used to set thresholds to select memory cells with an enriched Th1 cell gene-signature (>0.10) as well as a diminished Tfh cell gene-signature (<-0.05). **(f)** Relative enrichment of Tcm (left) and Tem (right) (Milnet et al., PNAS, 2020) (41) or **(g)** Tcmp (Ciucci et al., Immunity, 2019) (14) gene-signatures on uMAP plots generated from scRNA-seq of sorted SMARTA $CD4^+$ T cells over the course of an LCMV infection (day 7, 21, and 41).

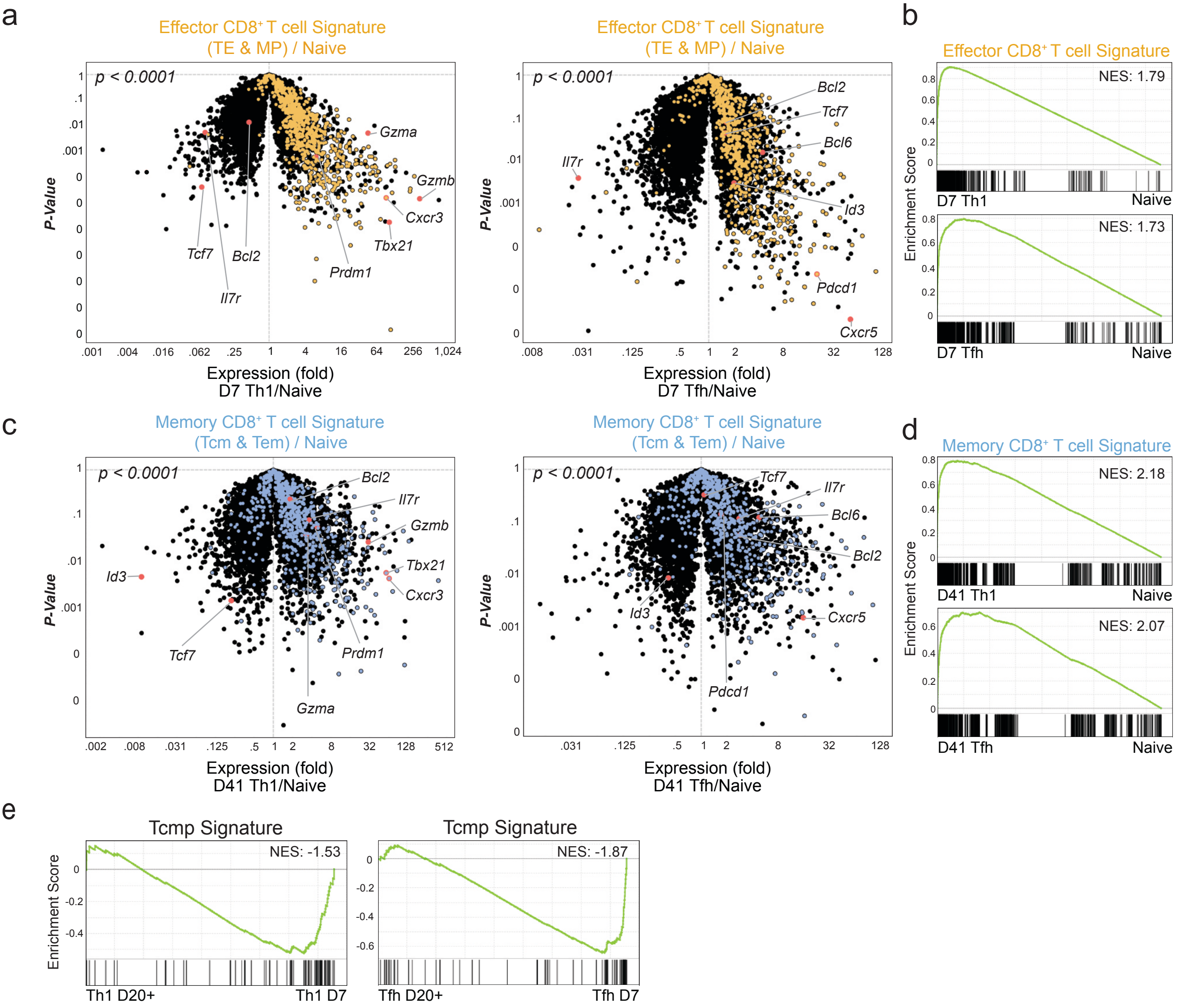
a
Effector CD8+ T cell Signature (TE & MP) / Naive
p < 0.0001
P-Value
Gzma
Gzmb
Cxcr3
Tbx21
Tcf7
Bcl2
Prdm1
Il7r
Expression (fold) D7 Th1/Naive
Effector CD8+ T cell Signature (TE & MP) / Naive
p < 0.0001
Bcl2
Tcf7
Bcl6
Il7r
Id3
Pdcd1
Cxcr5
Expression (fold) D7 Tfh/Naive
b
Effector CD8+ T cell Signature
NES: 1.79
D7 Th1
Naive
NES: 1.73
D7 Tfh
Naive
Enrichment Score
c
Memory CD8+ T cell Signature (Tcm & Tem) / Naive
p < 0.0001
Bcl2
Il7r
Gzmb
Tbx21
Cxcr3
Id3
Tcf7
Prdm1
Gzma
Expression (fold) D41 Th1/Naive
Memory CD8+ T cell Signature (Tcm & Tem) / Naive
p < 0.0001
Tcf7
Il7r
Bcl6
Bcl2
Id3
Cxcr5
Pdcd1
Expression (fold) D41 Tfh/Naive
d
Memory CD8+ T cell Signature
NES: 2.18
D41 Th1
Naive
NES: 2.07
D41 Tfh
Naive
Enrichment Score
e
Tcmp Signature
NES: -1.53
Th1 D20+
Th1 D7
Tcmp Signature
NES: -1.87
Tfh D20+
Tfh D7
Enrichment Score

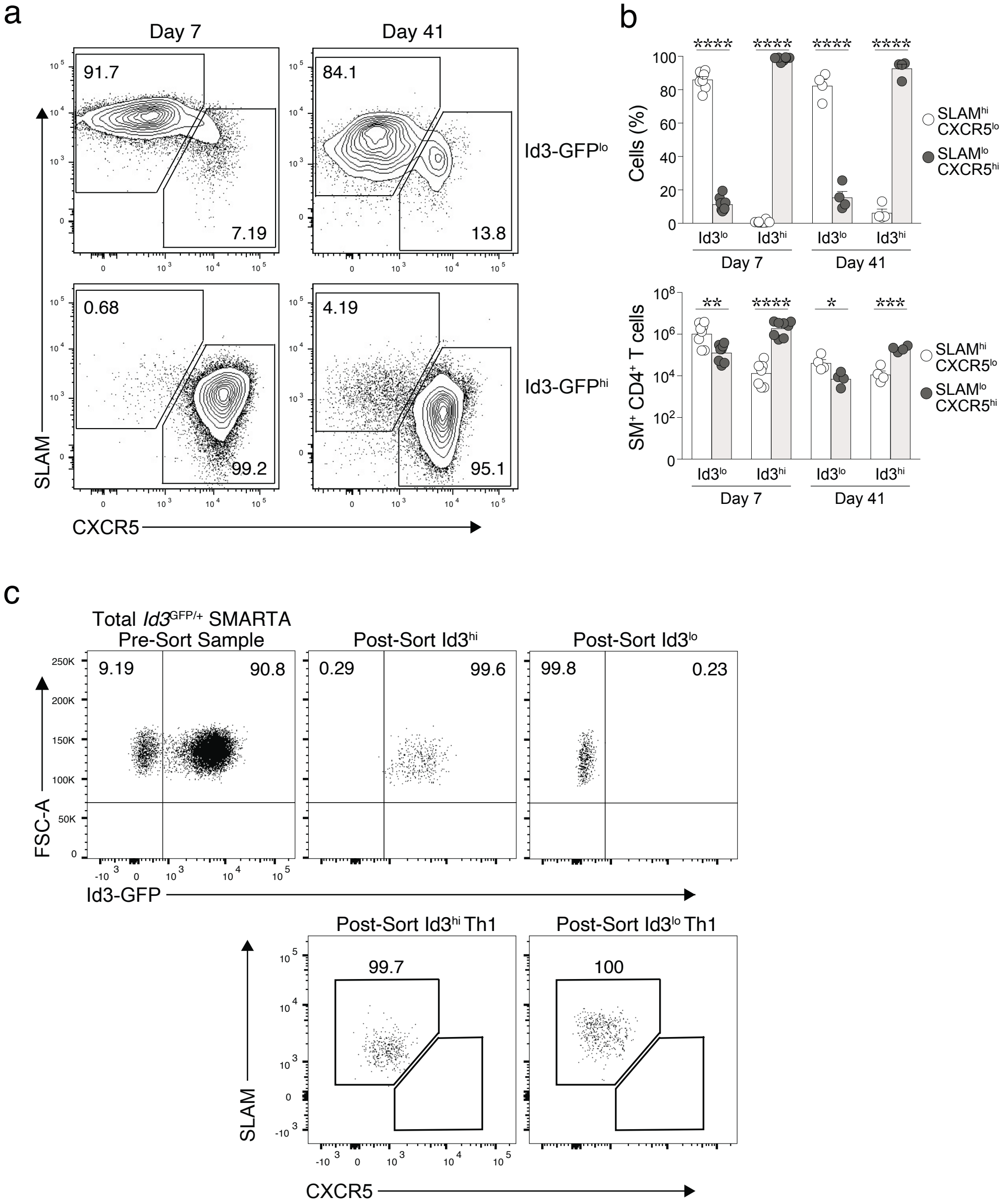
a
Day 7
Day 41
91.7
7.19
84.1
13.8
Id3-GFPlo
0.68
99.2
4.19
95.1
Id3-GFPhi
SLAM
CXCR5
b
Cells (%)
Id3lo
Id3hi
Day 7
Day 41
SLAMhi CXCR5lo
SLAMlo CXCR5hi
SM+ CD4+ T cells
c
Total Id3GFP/+ SMARTA Pre-Sort Sample
Post-Sort Id3hi
Post-Sort Id3lo
9.19
90.8
0.29
99.6
99.8
0.23
FSC-A
Id3-GFP
Post-Sort Id3hi Th1
Post-Sort Id3lo Th1
99.7
100
SLAM
CXCR5

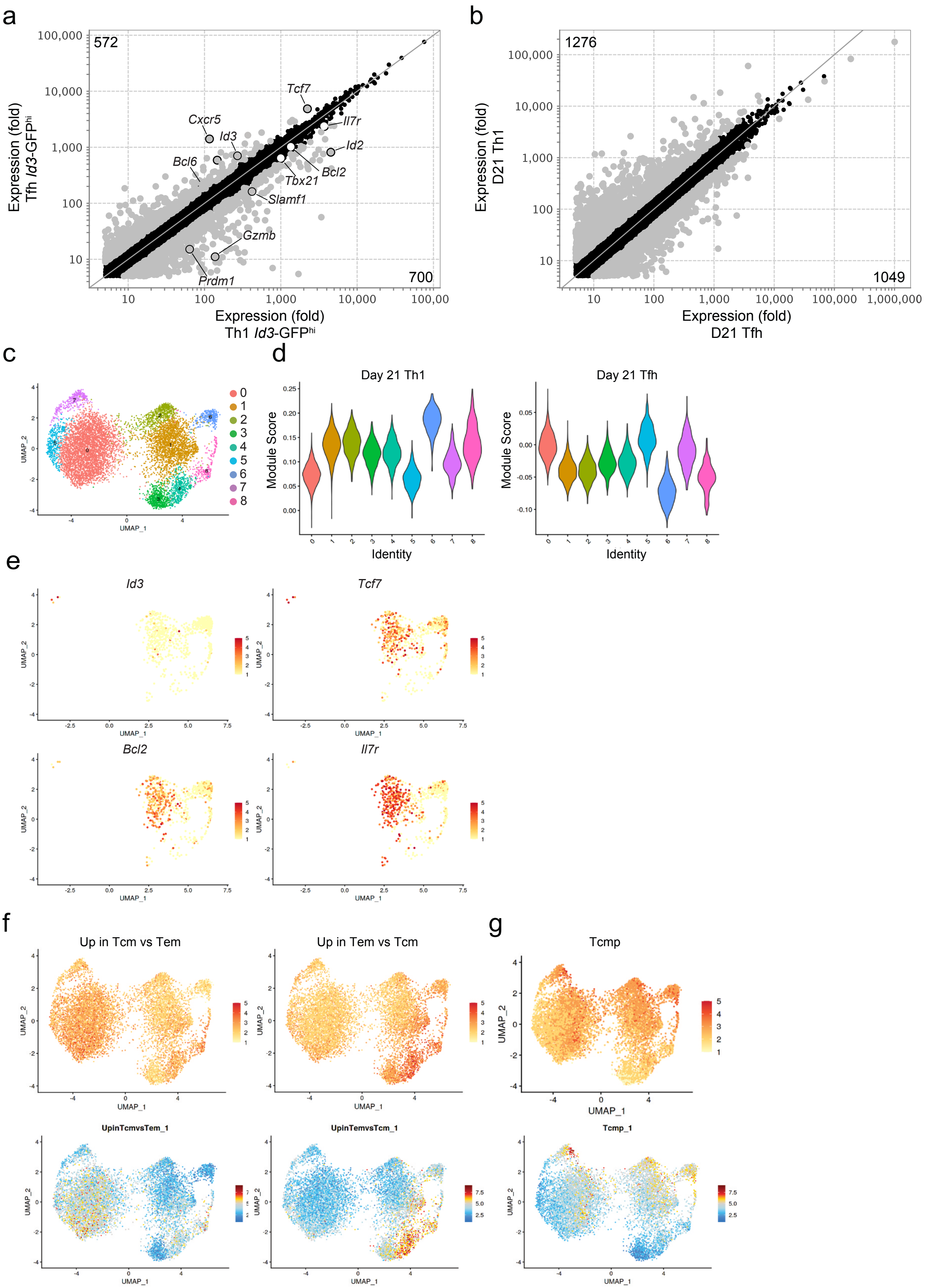

a
572
700
Tcf7
Cxcr5
Id3
Il7r
Id2
Bcl6
Bcl2
Tbx21
Slamf1
Gzmb
Prdm1
Expression (fold)
Tfh Id3-GFPhi
Expression (fold)
Th1 Id3-GFPhi
b
1276
1049
Expression (fold)
D21 Th1
Expression (fold)
D21 Tfh
c
UMAP_1
UMAP_2
d
Day 21 Th1
Day 21 Tfh
Module Score
Identity
e
Id3
Tcf7
Bcl2
Il7r
f
Up in Tcm vs Tem
Up in Tem vs Tcm
UpinTcmvsTem_1
UpinTemvsTcm_1
g
Tcmp
Tcmp_1
